# Sample Size Impact (*SaSii*): An R script for estimating optimal sample sizes in population genetics and population genomics studies

**DOI:** 10.1371/journal.pone.0316634

**Published:** 2025-02-13

**Authors:** Matheus Scaketti, Patricia Sanae Sujii, Alessandro Alves-Pereira, Kaiser Dias Schwarcz, Ana Flávia Francisconi, Matheus Sartori Moro, Kauanne Karolline Moreno Martins, Thiago Araujo de Jesus, Guilherme Brener Ferreira de Souza, Maria Imaculada Zucchi

**Affiliations:** 1 Biology Institute, State University of Campinas—UNICAMP, Campinas, São Paulo, Brazil; 2 Applied Biology Laboratory, Centro de Ensino Unificado do Distrito Federal, Brasília, Distrito Federal, Brazil; 3 Department of Genetics, Institute of Biological Sciences, Federal University of Amazonas, Manaus, Amazonas, Brazil; 4 Federal Institute of Education, Science and Technology of Brasília—Campus Recanto das Emas, Brasília, Distrito Federal, Brazil; 5 Department of Genetics, ESALQ/USP, Piracicaba, São Paulo, Brazil; 6 Secretariat of Agriculture and Food Supply of São Paulo State, APTA, UPDR-Piracicaba, São Paulo, Brazil; KGUT: Graduate University of Advanced Technology, ISLAMIC REPUBLIC OF IRAN

## Abstract

Obtaining large sample sizes for genetic studies can be challenging, time-consuming, and expensive, and small sample sizes may generate biased or imprecise results. Many studies have suggested the minimum sample size necessary to obtain robust and reliable results, but it is not possible to define one ideal minimum sample size that fits all studies. Here, we present *SaSii* (Sample Size Impact), an R script to help researchers define the minimum sample size. Based on empirical and simulated data analysis using *SaSii*, we present patterns and suggest minimum sample sizes for experiment design. The patterns were obtained by analyzing previously published genotype datasets with *SaSii* and can be used as a starting point for the sample design of population genetics and genomic studies. Our results showed that it is possible to estimate an adequate sample size that accurately represents the real population without requiring the scientist to write any program code, extract and sequence samples, or use population genetics programs, thus simplifying the process. We also confirmed that the minimum sample sizes for SNP (single-nucleotide polymorphism) analysis are usually smaller than for SSR (simple sequence repeat) analysis and discussed other patterns observed from empirical plant and animal datasets.

## Introduction

The study of genetic patterns at the population level interconnects ecology and evolution, thus providing a framework to understand the impact of selection, genetic drift, gene flow on demography, and phenotypic frequencies [1]. Spatial-temporal assessments, such as those applied in landscape genetics or using historic samples, can also be used to infer past population dynamics, human impacts in natural populations, and the effect of future environmental changes in populations [2]. In addition to the contributions of population genetics, genomic information is becoming increasingly available and accessible. Advances in genomic technologies have reduced the price per data point, expanding their impact on the development of basic and applied research with both model and non-model organisms [3].

All these applications and innovations require the development of methods to acquire and analyze data, and implementing all these technologies still requires a well-planned experimental design. Many publications over the last years have focused on describing molecular marker choice [3], protocol refinement [4, 5], overall process decisions [6–8], and sample sizes [9–11]. Poorly planned sample design and small sample sizes may prevent researchers from achieving robust results and conclusions and lead to biased parameter estimates [10, 11]. However, large sample sizes are often unfeasible due to time and budget reasons or limitations associated with the species or population characteristics, especially for endangered species. Thus, sample size is an aspect that has been debated by the scientific community and is frequently questioned by journal reviewers.

Although some studies have evaluated the effect of different sample sizes on data analysis and proposed sampling guidelines, there is no consensus on an ideal number that fits all studies. For microsatellite markers (SSR—Simple Sequence Repeats), the recommended minimum sample size ranged from 20 to over 40 individuals [9, 10]. For SNP (Single Nucleotide Polymorphism) data, the recommended minimum sample size ranged from eight to over 50 individuals [5, 11]. The variation results from differences in the hypothesis tested, the objectives of the studies, and the taxa evaluated. Therefore it is not possible to define a single ideal minimum sample size that fits all studies [12]. When empirical data is unavailable before the study begins, simulation studies and extrapolation from similar taxa may be helpful in decision-making. All sampling guidelines cited above were defined by data simulation and rarefaction curves. These methods are already well known by statisticians, but writing the scripts to perform the analyses may take time and effort, which can be very discouraging to some researchers.

We present *SaSii* (Sample Size Impact), a simulation program to help researchers define and indicate minimum sample size patterns for taxa groups. The patterns discussed here were obtained by analyzing simulated data and published datasets with *SaSii* and can be used as a starting point for the sample design of population genetics and genomic studies.

## Materials and methods

### About *SaSii*

**Fig 1:**
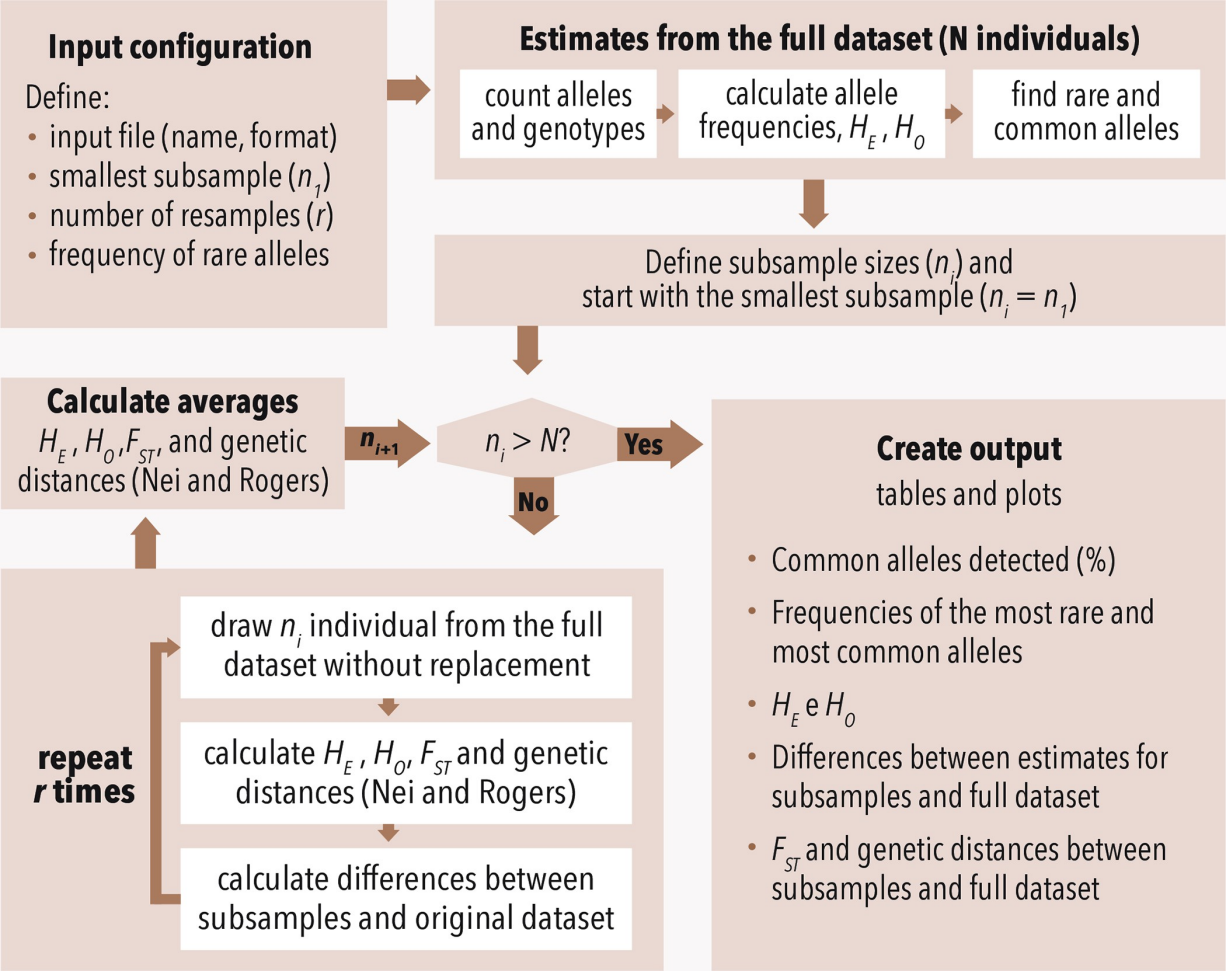
Script decision tree.

We introduce an R [13] script designed to assess whether the sample size is sufficient to estimate robust genetic parameters from SSR and SNP data. The minimum sample size is interpreted here as the number of individuals above which there is no more relevant gain in information for different genetic parameter estimates. This allows the user to define which parameters are relevant for each study. The program reads the input dataset that contains individuals genotyped at a certain number of markers, estimates genetic parameters from subsamples of different sizes defined by the user, and plots rarefaction curves (Fig 1). These estimates can be used to evaluate if the sample size is large enough to adequately represent the genetic diversity observed in the original full dataset, i.e. the rarefaction curve reaches a plateau or there is a small variance in the results from the subsample size. The script, tutorials, and other documentation are available at https://sasii.readthedocs.io/en/latest/index.html and in S1 File.

### Data input and config file

The program can read both SSR and SNP markers from multi-locus and diploid individuals’ data, formatted as a Structure [14] data file, accepting minor variations of the original format, and saved as a comma-separated values file (.csv). The individuals’ data are organized in rows and loci in columns. Alleles must be represented as numbers, with missing data coded as 0 (zero) or -9. The user may choose any of the two types of data organization adopted in Structure [14], one in which each individual is in one row and genotypes in two consecutive columns (the two alleles of the same locus are in two consecutive columns), and another in which each individual is in two consecutive rows and each locus in one column (the two alleles of the same locus are in two consecutive rows).

The user needs to fill a configuration file (hereafter *config* file) with parameters used to describe the data and the file structure. These parameters include the file name, input file organization, missing data code, and line number with the first individual of the population. The user also must define in this *config* file the following analysis settings: size of the smallest subsample, number of resamples of each subsample size and minimal frequency of allele to be preserved.

### Random subsampling of the dataset

The program makes random subsamples of the input dataset without replacement according to the settings defined by the user in the *config* file. For example, with a simulated dataset consisting of 50 individuals, with the smallest subsample size of five, the program will create new datasets with the following subsample sizes: 5, 10, 15, 20, 25, 30, 35, 40, 45, and 50. The smallest subsample size also defines the intervals between successive subsamples.

For each subsample, the program estimates genetic parameters and compares them to the original input dataset. It also calculates averages and standard deviations from the resamples of each subsample size and compares its estimates with the original dataset estimates to create the output tables and plots. The parameters estimated are: allele frequencies, observed heterozygosity (*H*_*O*_), expected heterozygosity under Hardy-Weinberg Equilibrium (*H*_*E*_), and genetic distances between the original dataset and the subsample (Wrigth’s *F*_*ST*_, Nei’s Distance, and Rogers’ distance). Parameters and equations are described in the S1 File.

### Data output and sample size impact analysis

The program outputs 14 pairs of files, each consisting of one numerical file and one plot with the results of the genetic parameter estimates, which are described briefly below. The researcher can use different parameters and, consequently different results from *SaSii* to define the minimum sample size, depending on the research question.

**Fig 2:**
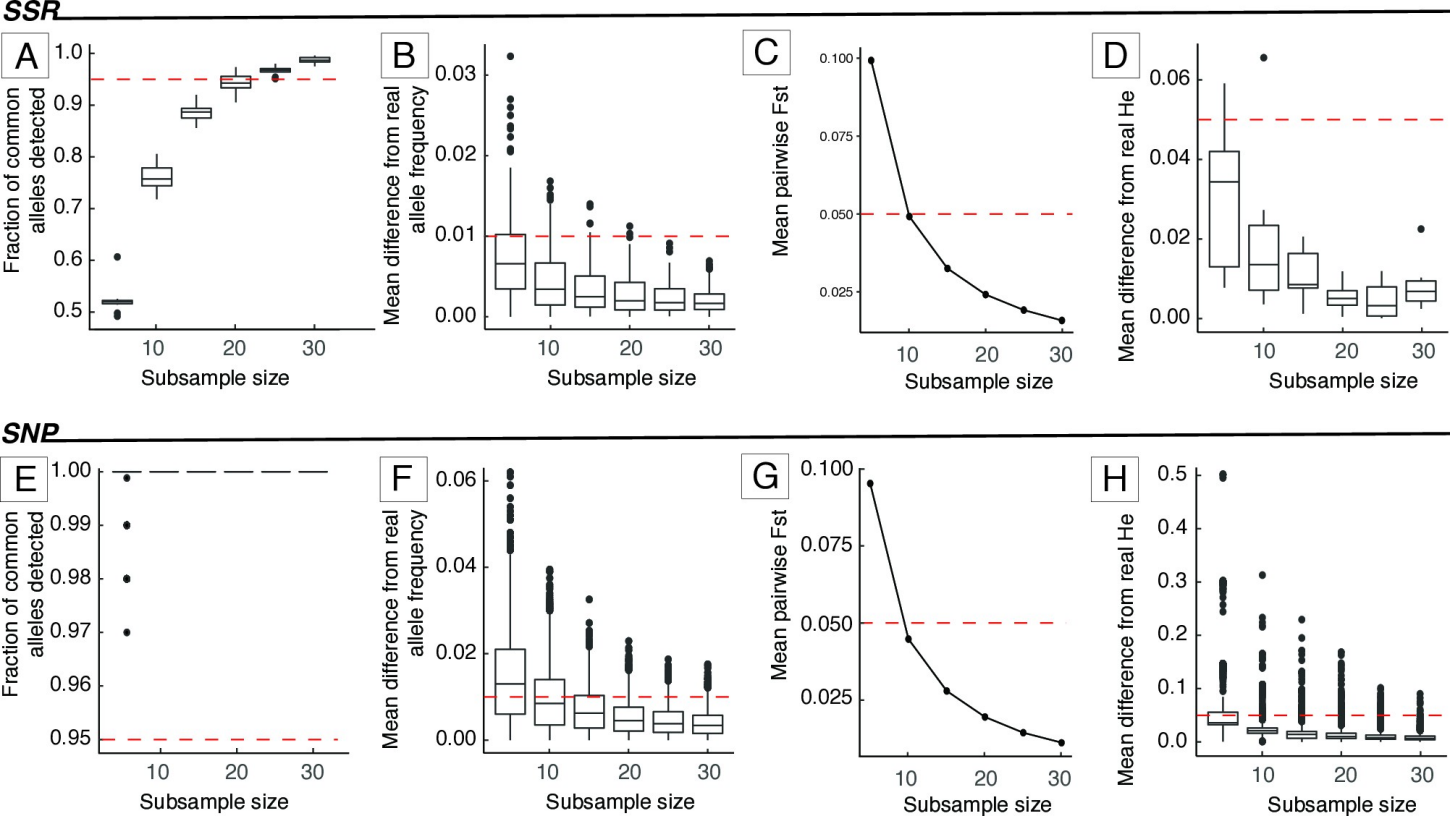
Results of *SaSii* analyses for SSR (top) and SNP (bottom) simulated data. Only the smallest subsample sizes are shown. A, E: Amount of individuals with more than 95% of alleles detected; B, F: mean difference of allele frequencies between the sampled populations and the original dataset; C, G: mean pairwise *F*_*ST*_ between the original and the simulated datasets; D, H: mean of expected heterozygosity of populations within a same class (same population and sample size).

*Fraction of common alleles detected (output 1)*. For some analyses, common alleles (frequency > 0.95) are more informative [10]. *SaSii* identifies the most common alleles in the input dataset and calculates their percentages in each subsample. Output 1 has a boxplot of the fraction of common alleles that are represented in the subsamples of each size (Fig 2A–2E). The red line in the plot indicates the threshold above which 95% of the common alleles are present in the subsamples. The red line in the plot indicates the threshold above which 95% of the common alleles are present in the subsamples. If detection of common alleles is important for the study, the subsample size that captures most or all common alleles may be interpreted as the minimum sample size. The rarefaction curve must reach a plateau close to the 0.95 line so we know that an increase in sample size will not have a large impact in the common allele number. If the curve does not reach a plateau, a larger sample size is necessary.

Outputs 2 and 3 have the same kind of results, but for the total number of alleles and only the rare alleles, respectively.

*Mean difference from original allele frequency (output 4)*. The program calculates the difference between the allele frequencies of the original dataset and the subsamples. The output table has the average difference for each allele for every subsample size. The output plot has a boxplot of these differences for each subsample size (Fig 2B and 2F). The red line indicates the threshold below which there is a 0.01 mean difference in allele frequencies from the original dataset and the subsample.

*Frequencies of the most and least common alleles (output 5)*. *SaSii* identifies the most and the least common alleles overall loci in the original dataset and estimates their frequencies in each subsample. The output table contains the mean allele frequencies, the standard deviations, and the minimum and maximum allele frequencies over subsamples. In addition, these results are also plotted in a boxplot, which contains a vertical line with the minimum and maximum values, a bold central line with the mean value, and a box with the standard deviations. The loss of rare alleles is usually one of the first indicators of bottlenecks [15], so small sample sizes should also show a deficit in rare alleles. Observing the frequency distribution of the least frequent alleles over different sample sizes may indicate the effect of sample reduction in allele detection.

*Expected heterozygosity (outputs 6 and 7)*. Genetic diversity analyses often require an accurate estimation of *H*_*E*_ in the population. So, for each sample size, *SaSii* calculates *H*_*E*_ (per locus in output 6 and averaged over all loci in output 7) of the simulated subsample and compares it to the estimate from the full dataset. In the tables of outputs 6 and 7, the user can find the mean *H*_*E*_ for each subsample size, the standard deviation over subsamples, and the minimum and maximum estimates over the subsamples. The output 6 plot is a boxplot of estimates for each subsample size. The output 7 plot contains a vertical line with the minimum and maximum *H*_*E*_ values, a bold central line with the mean value, and a box with the standard deviations.

*Observed heterozygosity (outputs 8 and 9)*. Outputs 8 and 9 are equivalent to outputs 6 and 7 but contain *H*_*O*_ estimates.

Mean difference from the original heterozygosities (outputs 10 and 11): Outputs 10 and 11 show the differences between *H*_*O*_ and *H*_*E*_ (respectively) from the original dataset and the subsamples. The output tables contain the mean difference over subsamples for each locus and subsample size. The output plots contain boxplots of the mean differences in heterozygosities over loci and subsamples for each subsample size. Fig 2D and 2H show the plots for HE. The red line in the plots indicates greater deviances than 0.05.

*Mean pairwise F*_*ST*_ *and genetic distances (outputs 12*, *13*, *and 14)*. *SaSii* calculates the genetic differentiation (*F*_*ST*_ [16]), Nei’s distance [17], and Rogers’ distance [18] between subsamples and the original dataset to check if the subsamples are giving accurate and consistent results which may be indicated by small genetic distances [10]. Mean values over loci and standard deviation are estimated and presented in the output tables. The output plots show the average values over subsamples for each subsample size. The *F*_*ST*_ plot has a red line with the 0.05 threshold, which indicates low population genetic differentiation (Fig 2C–2G).

### Sample size impact analysis

To estimate the minimum sample size, we suggest that the users analyze the predefined threshold for each plot. All sample sizes that pass these thresholds can be considered large enough for that parameter. When different parameters indicate different minimum sample sizes, we suggest a conservative approach, selecting the largest size as the overall minimum. For a better analysis, we suggest at least four graphs: outputs 1, 4, 12, and 12, that summarize most of the genetics parameters for the species.

### Script validation

To evaluate *SaSii*’s predictive accuracy, we constructed two datasets using EasyPop 2.0.1, a forward simulator, to generate similar data to those found in nature [19]. These populations were intended to simulate SSR and SNP markers, using the following parameters: ploidy (2), sex number (2), mating system (Random), number of populations (1), population size (600), male and female ratio (1:1), free recombination (yes), same mutation scheme (yes), mutation model (mixed model of SSM—stepwise model—with a proportion of KAM—K-allele model—mutation events), proportion of KAM events (0.5), variability of initial population (maximal) and number of generations (10). The number of loci, mutation rate, and number of possible allelic states were different for SSR and SNP markers, respectively: 10 and 2,500 loci, 1 × 10^−4^ and 1 × 10^−7^ for mutation rates, and 20 and two allelic states.

Then, we configured *SaSii*’s input setting the default value for all common parameters (resampling number, sample size, minimum frequency, and max sample size) for both simulated datasets. *SaSii* generated fifty subsamples from the amount of each population’s size, with a sample size equal to five, selecting allele frequency with at least 0.05. The remaining parameters were defined depending on the data file of each species. All datasets were analyzed in a workstation with the following configuration: AMD Ryzen 7 5700X, 32GB RAM. The script was also tested on personal computers with different operating systems (Linux, MacOS, Windows).

### Patterns to predict the minimum sample size

We investigated if any characteristics of the species or the type of molecular marker could be used as a predictor for generalizations about the minimum sample size necessary for genetic estimates. We used empirical datasets from population genetic studies: one insect, one reptile, and 15 plant species (16 populations), of which seven were SSR and eight were SNP data as shown in Table 1. The results found here for each species may apply to other studies and improve the decision-making process in population genetic studies. *SaSii* runs for all species followed the same settings as the validation analyses.

**Table 1:** Characteristics of the datasets used to estimate minimum sample size.

Category	Species	Mating system	Sample size	Number of loci	Reference
Plant SNP	*Acrocomia aculeata*	Mixed	25	3,269	[20]
	*Casearia sylvestris*	Allogamy	23	1,257	[21]
	*Chloropyron maritimum*	Allogamy	15	111	[22]
	*Euterpe precatoria*	Allogamy	12	3,803	Francisconi et al. (work is in progress)
	*Manihot dulcis*	Allogamy	46	1,985	[23]
	*Manihot esculenta*	Allogamy	53	1,985	[23]
Plant SSR	*Bertholletia excelsa*	Allogamy	91	12	[24]
	*Caryocar villosum*	Allogamy	38	7	[25]
	*Copaifera langsdorffii*	Mixed	357	8	[26]
	*Erythrophleum suaveolens* (pop 1)	Mixed	177	9	[26]
	*Erythrophleum suaveolens* (pop 2)	Mixed	88	9	[27]
	*Eugenia dysenterica*	Allogamy	28	7	[28]
	*Metrosideros polymorpha*	Allogamy	319	9	[29]
	*Myroxylon peruiferum*	Mixed	50	9	[30]
	*Shorea macrophylla*	Mixed	88	14	[31]
	*Solanum lycocarpum*	Allogamy	178	5	[32]
Animal SNP	*Tetragonisca angustula*	-	32	3,573	[33]
Animal SSR	*Caiman crocodilus*	-	25	11	[34]

Here, the minimum sample size was defined based on the proportion of common alleles detected, the mean difference of allele frequencies, the mean pairwise *F*_*ST*_, and the mean difference in *H*_*E*_. We tested the null hypothesis of no differences in minimum sample size for datasets of SNP and SSR markers using all plant and animal datasets. We also tested the null hypothesis of no difference in minimum sample size for mating systems in allogamous and mixed mating plant datasets. We used a two-sided Wilcoxon rank sum test in R [13] to perform the statistical test.

## Results

### Script validation

The main results from the analyses performed with *SaSii* with the simulated dataset are shown in Fig 2. For the SSR data, the minimum sample size ranged from 10 to 45, depending on the parameter analyzed (Fig 2A–2D). In Fig 2A and 2C, for subsamples with a minimum of 10 individuals, at least 95% of common alleles were detected, and the mean *F*_*ST*_ was smaller than 0.05. However, in the plots that presented the differences between subsamples and the full dataset in allele frequencies and *H*_*E*_ (Fig 2B and 2D), the minimum sample size was 15 and 45 individuals, respectively. For larger subsamples, there was no relevant increase in precision, so the results are not shown.

Using the same strategy for the SNP data, the plots suggested that five individuals were necessary to have a minimum of 95% of alleles detected (Fig 2E), and a low mean difference in *H*_*E*_ (Fig 2H). Meanwhile, 10 and 15 individuals were sufficient for a *F*_*ST*_ smaller than 0.05 (Fig 2G), and a low mean difference allele frequency (Fig 2F).

### Patterns to predict the minimum sample size

**Fig 3:**
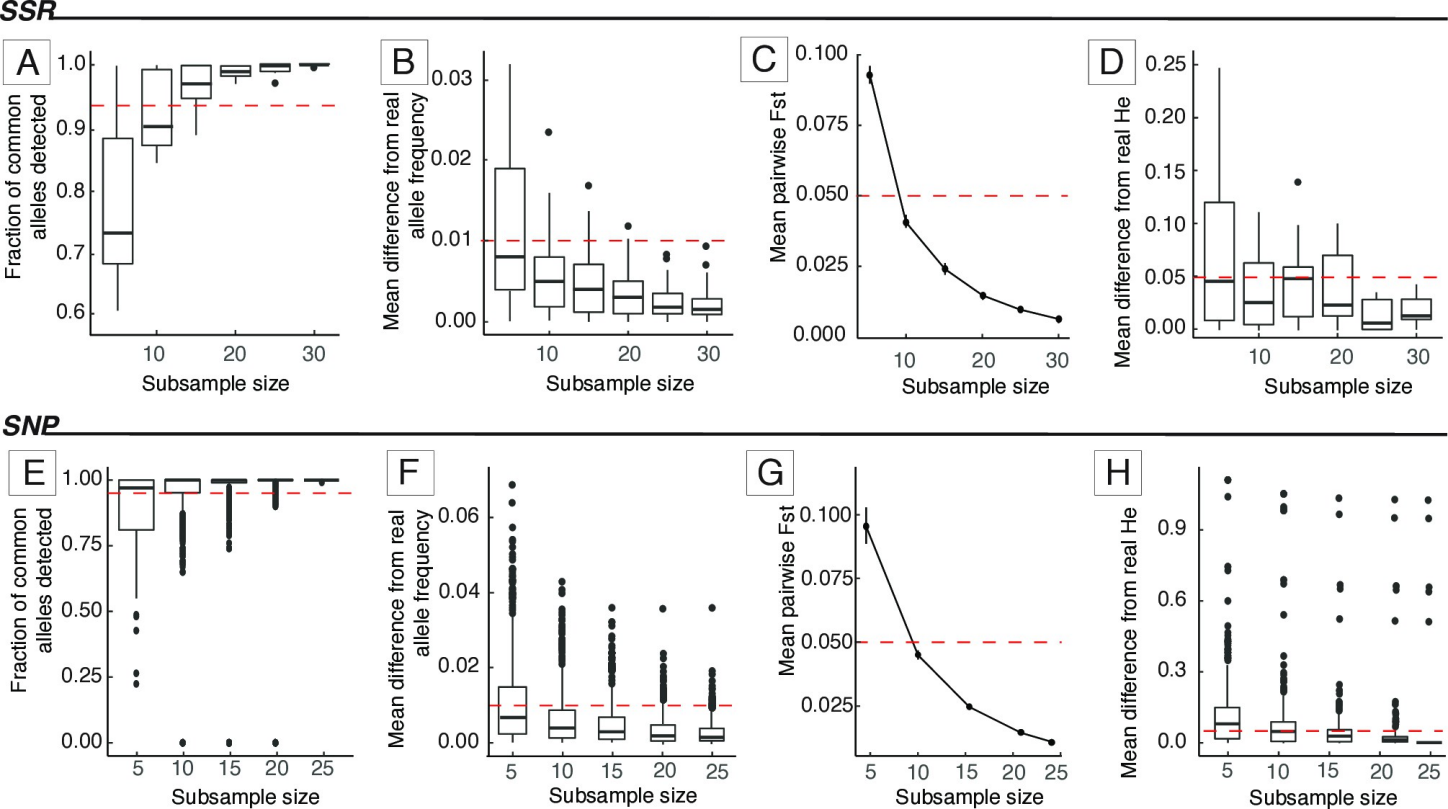
Results of *SaSii* analyses for *Bertholletia excelsa* and *Casearia sylvestris* datasets. A,E: proportion of common alleles detected in each subsample; B,F: mean difference of allele frequency between the sampled populations and the empirical dataset; C,G: mean pairwise *F*_*ST*_ between subsample and complete dataset; D,H: mean difference in *H*_*E*_ estimates between subsamples and the complete dataset.

We analyzed data from species of plants and animals and observed a minimum sample size ranging from five to 45 (Table 2). All results files can be found at https://sasii.readthedocs.io/en/latest/index.html. We observed that the minimum sample size could differ for each species depending on the parameter analyzed. For example, we identified that for *Bertholletia excelsa*, a species evaluated with SSR markers (91 samples, 12 loci), 15 individuals were sufficient to detect at least 95% of the common alleles and small mean differences from the original *H*_*E*_ (< 0.05; Fig 3A and 3B). Subsamples of 10 or more individuals presented *F*_*ST*_ values smaller than 0.05 when compared with the full dataset (Fig 3C), however, when considering the allele frequencies only 10 individuals were sufficient to achieve a small difference from the original data (Fig 3D).

**Table 2:** Minimum sample sizes detected with *SaSii*.

Category	Species	Common alleles	Allele freq.	*F*_*ST*_	*H*_*E*_	Overall
Plant SNP	*Acrocomia aculeata*	10	5	5	10	10
	*Casearia sylvestris*	10	10	10	20	20
	*Chloropyron maritimum*	5	5	10	5	10
	*Euterpe precatoria*	10	10	15	10	15
	*Manihot dulcis*	5	10	10	15	15
	*Manihot esculenta*	10	10	10	20	20
Plant SSR	*Bertholletia excelsa*	15	10	10	15	15
	*Caryocar villosum*	15	5	10	15	15
	*Casearia sylvestris*	40	5	10	10	40
	*Copaifera langsdorffii*	20	5	10	10	20
	*Erythrophleum suaveolens* (pop 1)	20	10	10	25	25
	*Erythrophleum suaveolens* (pop 3)	20	5	10	15	20
	*Eugenia dysenterica*	15	10	10	10	15
	*Metrosideros polymorpha*	25	5	10	20	25
	*Myroxylon peruiferum*	20	15	10	25	25
	*Shorea macrophylla*	20	5	10	15	20
	*Solanum lycocarpum*	15	5	10	20	20
Animal SNP	*Tetragonisca angustula*	5	10	10	10	10
Animal SSR	*Caiman crocodilus*	15	10	10	15	15

Sample sizes selected considering different parameters. Fraction of common alleles detected (Common alleles), mean difference from the complete dataset allele frequency (Allele freq.), mean pairwise *F*_*ST*_ between each subsample and the full dataset (*F*_*ST*_), mean difference from the full dataset *H*_*E*_, and the largest value from all parameters (Overall).

For SNP data, the minimum sample size was smaller than SSR. We show the analysis of *Casearia sylvestris* (23 samples, 1,257 loci) as an example (Fig 3E–3H). For this species, with only five individuals we obtained a good representation of the original data, as shown by the fraction of common alleles detected and the mean difference of allele frequencies (Fig 3E and 3F). However, subsamples with less than 10 individuals showed higher *F*_*ST*_ and larger mean *H*_*E*_ differences from the original dataset (Fig 3G and 3H), suggesting the need for additional individuals for more robust results. Considering this, the minimum sample size may range between five and 15 individuals, depending on the criterion adopted and the objectives of the study.

**Fig 4:**
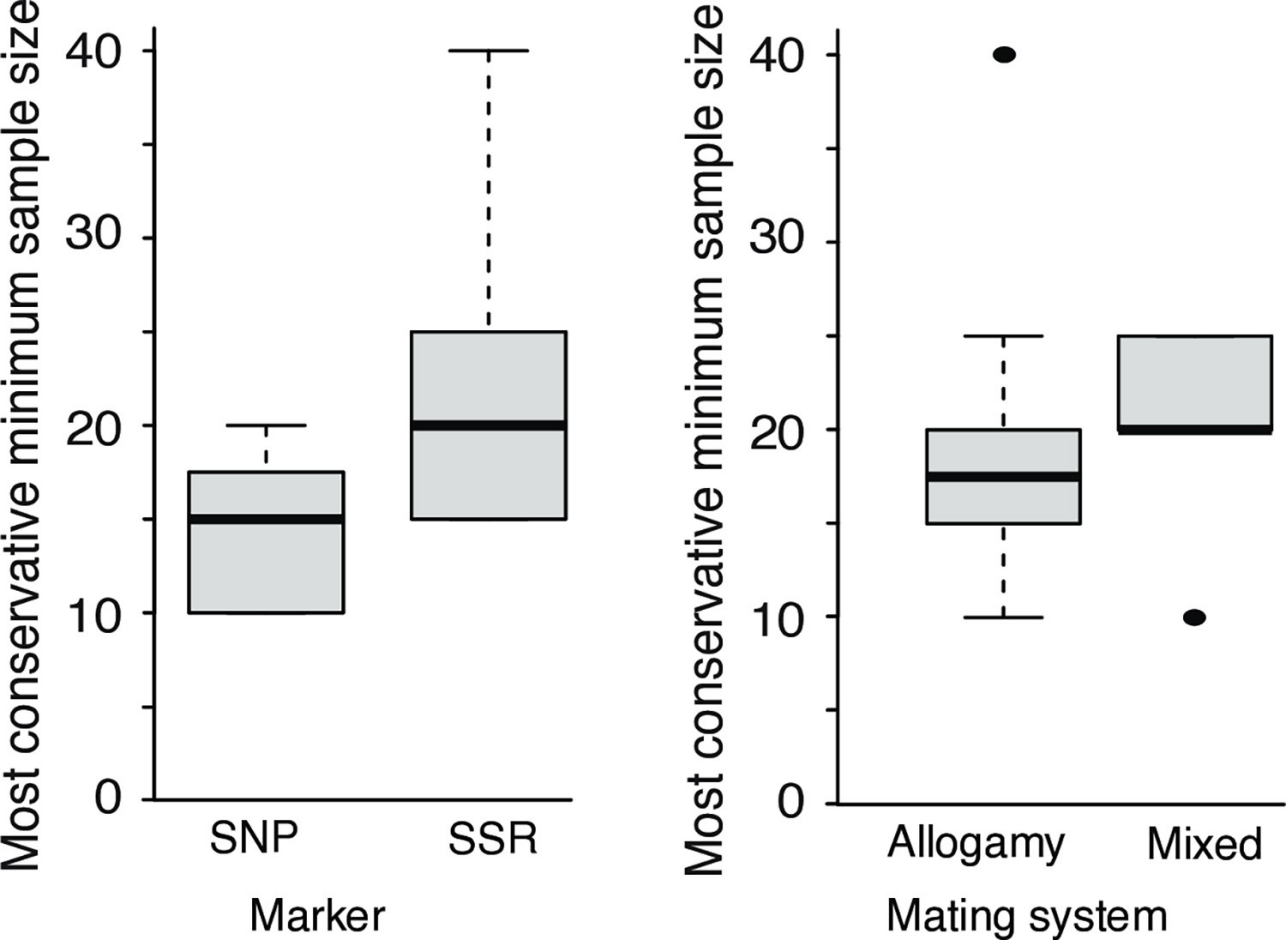
Most conservative minimum sample sizes observed for empirical datasets grouped by molecular marker type (left) and mating system (right).

The most conservative minimum sample size found for SSR markers was between 15 and 40 individuals. For SNP datasets, the majority overall minimum sample size ranged from 10 to 20 individuals, being influenced by the *F*_*ST*_ estimated between the complete dataset and each subsample. In most cases, we observed that minimum sample sizes were smaller for SNP than for the SSR dataset (W = 16, p = 0.026) (Fig 4 left). This pattern was also observed for *C*. *syslvestris* for which we analyzed samples from the same area genotyped with both markers (Fig 4). When we compared minimum sample sizes for plants with different mating systems, there was no significant difference (W = 23, p = 0.464) (Fig 4 right).

## Discussion

*SaSii* script was efficient in analyzing both SSR and SNP datasets, using computers with standard configurations, and different operating systems (Linux, MacOS, Windows). Our results showed that the minimum sample sizes varied between analyzed species and depended on the parameters evaluated. Overall, for SNP data, a smaller sample size was necessary to obtain robust results when compared to SSR data results. The script presented here can be used both to help develop sample strategies and to evaluate if the samples collected are sufficient for the analyses.

The analysis of simulated data indicated that *SaSii* can provide precise estimations of *H*_*O*_ and *H*_*E*_. Larger subsamples show values for these parameters similar to the total dataset and smaller subsamples show higher variance in estimates. For SSR simulated data, 25 individuals were enough to represent the full dataset. For SNP data, the minimum sample size was 10 individuals. It is well known that the sample size for SSR and SNP data are around 25 to 40 and eight to 50, respectively [5, 9–11]. This number may vary depending on the species, location, and study focus. Our simulation results confirm these recommendations.

As for our empirical SSR datasets, we found that, for some populations and species, the minimum sample size was smaller than the suggested in the literature, for others, *SaSii* results indicated the same as the literature [9, 10]. For empirical SNP data, our results were similar to the literature suggestions [5, 11]. This highlights the importance of taking the particularities of each species into account. Using information on similar species can be useful for initial experimental design, but checking if the sample was large enough increases the chance of having robust results. Also, it is important to consider sampling design, because random population-wide sampling has the potential to include a broader genetic diversity than sampling individuals in clusters or a limited portion of the population distribution. In these cases, the results shown by the program may be biased.

Our results suggest that most SSR and SNP data usually presented similar sample size requirements, within the marker’s type. Patterns for *C*. *crocodilus* and *T*. *angustula* differ from the other datasets probably because they are both animal species, which can have different characteristics that can impact the minimum sample size. Besides that, in plants, factors such as pollination syndrome, seed dispersal, and population characteristics (for example, density, genetic structure, allelic richness, and effective population size) can influence the minimum sampling size [11]. For *C*. *langsdorffii*, high levels of inbreeding, which resulted in small estimates of observed heterozygosity, were reported in different genetic studies of natural populations [35, 36]. The empirical dataset for this species may reflect this condition, which resulted in a larger sample size than for the other species that used the same type of marker. It is important to understand that the minimum sample size estimated here represents the smallest number of individuals that can be used to precisely estimate genetic parameters. So, *SaSii* should be used as a study design tool to define the sample size necessary to obtain robust population genetic estimates and to evaluate if the obtained sample size was large enough. Other studies discuss and propose methodologies to estimate the minimum sample size and sampling strategies for collecting germplasms and ex-situ conservation [37–41], and ecological restoration [42].

After getting *SaSii* outputs, we evaluated if the marker type and the mating system could influence the minimum sample size. This turned out to be true for marker type: as suggested by previous studies, when using SNP markers lower sampling sizes than SSR markers would be required to represent the species’ genetic diversity. However, we did not find a significant difference between minimum sample size in plants with different mating systems. We believe that *SaSii* can aid future genetic researchers providing tools for simulating populations and plotting graphics that summarize the most important parameters for population genetics studies. From our simulated and empirical data output, we propose that it is possible to reduce even more the sample sizes from SSR and SNP data, reducing genetics studies costs and putting less pressure on endangered species.

*SaSii* requires a dataset with genotyped samples to run. If the user wants to verify whether their sample is large enough, the dataset is already available. However, if *SaSii* is used as a sample design tool, this data is probably not yet available. To overcome this limitation, the input data may be a dataset from a similar taxon (e.g. another population from the same species, species with similar population size, demographic history, and other parameters that may influence the variance in genetic diversity). Another option is to create a simulated dataset, as performed here for the program validation, setting simulation parameters to match the target species’ characteristics. The current script was designed to analyze only diploid data. Therefore, the script should not be used for haploid or polyploid species, unless the user makes the necessary changes to the script. Our tests also showed a limitation on memory usage for R in datasets with large numbers of markers or individuals. We recommend that *SaSii* should be used in personal computers to analyze genomic data with up to 15,000 SNPs and with at most 300 individuals. To analyze larger datasets, the user should have access to a better workstation.

## Conclusion

We presented *SaSii* an R script that takes SNP and SSR data to calculate estimates of genetic diversity and provide plots as outputs to help researchers decide a reasonable sampling effort for their genetic studies. Our results showed that it is possible to estimate an adequate sample size that represents populations and does not require that the scientist take time to write any program code, extract and sequence samples, or use genetics population programs, making it easier to gather this information. Our results showed that a sample size per population of five to 25 for SNP and 15 to 30 for SSR could be used for most plant species, giving a better direction for new studies.

### Data accessibility

The script, tutorials, other documentation, and simulated data are available at https://sasii.readthedocs.io/en/latest/index.html. Each empirical dataset availability is described in its corresponding article.

## Supporting information

S1 File Zipped file containing: (A) A file containing more detailed information on the parameters estimated by *SaSii*, including the equations used for the calculations, and with examples of accepted input formats. (B) *SaSii* script. (C) Configuration file with parameters used to describe the dataset and analysis settings.
(ZIP)
