# Supplementary material for: Sample Size Impact (SaSii): An R script for estimating optimal sample sizes in population genetics and population genomics studies
Source: PLoS One. 2025 Feb 13;20(2):e0316634. doi: 10.1371/journal.pone.0316634 (PMC11824989; doi:10.1371/journal.pone.0316634)
Supplement: S1 File — Zipped file containing: (A) A file containing more detailed information on the parameters estimated by SaSii, including the equations used for the calculations, and with examples of accepted input formats. (B) SaSii script. (C) Configuration file with parameters used to describe the dataset and analysis settings. (ZIP) [file pone.0316634.s001.zip › Supplemental_Information_1.pdf]

Supplemental Information for:

Sample Size Impact (*SaSii*): an R script for estimating optimal sample sizes in population genetics and population genomics studies

Matheus Scaketti, Patricia Sanae Sujii, Alessandro Alves-Pereira, Kaiser Dias Schwarcz, Ana Flávia Francisconi, Matheus Sartori Moro, Kauanne Karolline Moreno Martins, Thiago Araujo de Jesus, Guilherme Brener Ferreira de Souza, Maria Imaculada Zucchi

What does *SaSii* do?

1. Resampling

SaSii does random subsamples from the input data. The subsamples size varies from a minimum size (n) to the number of individual present in the input file, and the intermediate classes will have sizes corresponding to multiples of n. The number of repetitions for each subsample size is any integer number greater than 100.

2. Original input sample and subsamples parameter estimation

- Allele frequencies (p_i);
- Expected heterozygosity under Hardy-Weinberg equilibrium (H_E) per locus and overall;
- Observed heterozygosity (H_O) per locus and overall.

3. Subsamples repetitions parameter estimation

- Mean H_E, variance and standard deviation (*SD*) for the repetitions per locus per subsample size;
- Mean H_E overall loci per subsample size.

3. Rare alleles

- Identify alleles from the original input sample with frequencies lower than 5% ($p_i < 0.05$);
- For each subsample size, calculate the proportion of repetitions which have all alleles from the input sample with frequencies equal to or higher than 0.05.

4. Differences between input sample and subsamples

- For each allele, estimates difference between allele frequency of the input sample (p_{pi}) and the mean allele frequency from the repetitions of each subsample size ($\overline{p_{si}}$), and the variance;

$$\Delta\overline{p_i} = |p_{pi} - \overline{p_{si}}|$$

- Pairwise F_{ST} (Wright 1949) between input sample and each subsample;
- Mean pairwise F_{ST} and SD for the repetitions of each sample size;

$$F_{ST} = \frac{H_T - H_S}{H_T}$$

$$H_T = 1 - \Sigma (p_i)^2$$

$H_S = \Sigma (H_E/n_p)$, n_p is the number of populations being compared.

- Nei's genetic distance (Nei 1972) between input sample and each subsample;
- Mean pairwise Nei's distance and SD for the repetitions of each sample size;

$$D = -\ln (I)$$

$$I = \frac{\Sigma (x_i\, y_i)}{\sqrt{j_x\, j_y}}$$, xi and yi are the allele frequencies in each population

$j_x = \Sigma\, x_i^2$ $j_y = \Sigma\, y_i^2$

- Rogers-Wright distance (1978) between input sample and each subsample;
- Mean pairwise Rogers-Wright distance and SD for the repetitions of each sample size;
  $D_{12} = \sqrt{}\, (1/2L)\, \Sigma_l\, \Sigma_i\, (p_{li1} - p_{li2})$, L is the number of loci; p_{li1} and p_{li2} are the frequency of allele 1 and allele 2 from each locus (l), respectively;

Results

	Locus1		Locus2		Locus3	
Ind1	230	238	101	103	138	146
Ind2	230	238	101	103	146	146
Ind3	224	238	0	0	146	146
Ind4	238	238	0	0	146	146
Ind5	238	238	101	103	146	146

;Locus1;;Locus2;;Locus3;
Ind1;230;238;101;103;138;146
Ind2;230;238;101;103;146;146
Ind3;224;238;0;0;146;146
Ind4;238;238;0;0;146;146
Ind5;238;238;101;103;146;146

Figure S1. Example of an input file expected in SaSii, with individuals coded in one row. In this example, five individuals were genotyped with four microsatellite loci, and the alleles of a single locus are represented as the number of the amplified fragment in two consecutive rows. In this example, missing data was coded as zero. Left: Dataset as observed in a spreadsheet. Right: Dataset as observed in text form.

	Locus1	Locus2	Locus3
Ind1	230	101	138
Ind1	238	103	146
Ind2	230	101	146
Ind2	238	103	146
Ind3	224	0	146
Ind3	238	0	146
Ind4	238	0	146
Ind4	238	0	146
Ind5	238	101	146
Ind5	238	103	146

;Locus1;Locus2;Locus3;
Ind1;230;101;138
Ind1;238;103;146
Ind2;230;101;146
Ind2;238;103;146
Ind3;224;0;146
Ind3;238;0;146
Ind4;238;0;146
Ind4;238;0;146
Ind5;238;101;146
Ind5;238;103;146

Figure S2. Example of an input file expected in SaSii, with individuals coded in one row.
In this example, five individuals were genotyped with four microsatellite loci, and the alleles of a single locus are represented as the number of the amplified fragment in two consecutive rows. In this example, missing data was coded as zero. Left: Dataset as observed in a spreadsheet. Right: Dataset as observed in text form.

References

Nei, M. (1987). Genetic distance between populations. In Molecular evolutionary genetics (pp. 208-253). Columbia University Press. https://doi.org/10.7312/nei-92038-010

Roger, J. S. (1972). Measure of genetic similarity and genetic distance. Studies in genetics VII. University of Texas publication, 7213, 145-153.
Wright, S. (1949). The genetical structure of populations. Annals of eugenics, 15(1), 323-354. https://doi.org/10.1111/j.1469-1809.1949.tb02451.x
